# Prognostic factors of adult tuberculous meningitis in intensive care unit: a single-center retrospective study in East China

**DOI:** 10.1186/s12883-021-02340-3

**Published:** 2021-08-10

**Authors:** Baobao Feng, Xiao Fei, Ying Sun, Xingguo Zhang, Deya Shang, Yi Zhou, Meiyan Sheng, Jiarui Xu, Wei Zhang, Wanhua Ren

**Affiliations:** 1 Department of Emergency, Cheeloo College of Medicine, Shandong Provincial Hospital, Shandong University, 250021 Jinan, Shandong China; 2 Department of Emergency, Shandong Provincial Hospital Affiliated to Shandong First Medical University, 250021 Jinan, Shandong China; 3 Department of Infectious Diseases, Weifang Yidu Central Hospital, 262500 Weifang, Shandong China; 4 Department of Critical Care Medicine, Cheeloo College of Medicine, Shandong Provincial Chest Hospital, Shandong University, 250013 Jinan, Shandong China; 5 Department of Infectious Diseases, Cheeloo College of Medicine, Shandong Provincial Hospital, Shandong University, 324 Jingwu Weiqi Road, 250021 Jinan, Shandong China; 6 Department of Infectious Diseases, Shandong Provincial Hospital Affiliated to Shandong First Medical University, 250021 Jinan, Shandong China

**Keywords:** Tuberculous meningitis, Prognostic factors, Intensive care unit

## Abstract

**Background:**

Tuberculous meningitis (TBM) is the most lethal form of tuberculosis worldwide. Data on critically ill TBM patients in the intensive care unit (ICU) of China are lacking. We tried to identify prognostic factors of adult TBM patients admitted to ICU in China.

**Methods:**

We conducted a retrospective study on adult TBM in ICU between January 2008 and April 2018. Factors associated with unfavorable outcomes at 28 days were identified by logistic regression. Factors associated with 1-year mortality were studied by Cox proportional hazards modeling.

**Results:**

Eighty adult patients diagnosed with TBM (age 38.5 (18–79) years, 45 (56 %) males) were included in the study. An unfavorable outcome was observed in 39 (49 %) patients and were independently associated with Acute Physiology and Chronic Health Evaluation (APACHE) II > 23 (adjusted odds ratio (aOR) 5.57, 95 % confidence interval (CI) 1.55–19.97), Sequential Organ Failure Assessment (SOFA) > 8 (aOR 9.74, 95 % CI 1.46–64.88), and mechanical ventilation (aOR 18.33, 95 % CI 3.15–106.80). Multivariate Cox regression analysis identified two factors associated with 1-year mortality: APACHE II > 23 (adjusted hazard ratio (aHR) 4.83; 95 % CI 2.21–10.55), and mechanical ventilation (aHR 9.71; 95 % CI 2.31–40.87).

**Conclusions:**

For the most severe adult TBM patients of Medical Research Council (MRC) stage III, common clinical factors aren’t effective enough to predict outcomes. Our study demonstrates that the widely used APACHE II and SOFA scores on admission can be used to predict short-term outcomes, while APACHE II could also be used to predict long-term outcomes of adult patients with TBM in ICU.

**Supplementary Information:**

The online version contains supplementary material available at 10.1186/s12883-021-02340-3.

## Background

Tuberculosis is one of the top 10 causes of death, and the leading cause from a single infectious agent worldwide [1]. Tuberculous meningitis (TBM) is the most lethal form of tuberculosis, which accounts for approximately 1 % of all cases of active tuberculosis, and 5–10 % of extra-pulmonary tuberculosis cases [2, 3]. Mortality in adult patients with TBM reaches 30–60 % [4], and neurological sequelae were reported in more than 50 % of survivors [5]. Delayed diagnosis and treatment, higher Medical Research Council (MRC) disease severity stage, lower cerebrospinal fluid (CSF) lymphocyte cell count, and anti-tuberculosis drug resistance were reported to be associated with an unfavorable outcome in previous studies [6, 7]. Patients with TBM who had neurological complications frequently require admission to the intensive care unit (ICU) [8, 9]. However, studies on patients with TBM requiring ICU admission are scarce due to the limited access of patients to intensive care in developing countries. Therefore, we conducted this retrospective study on adult patients with TBM admitted to ICU in a tuberculosis endemic area. Our objective was to identify prognostic factors of unfavorable outcome in adult patients with TBM admitted to the ICU.

## Methods

### Design, setting, and participants

This was a single-center retrospective study on consecutive adult patients with TBM admitted to the medical ICU of Shandong Provincial Chest Hospital, Cheeloo College of Medicine, Shandong University, a 900-bed tertiary hospital located in Shandong Province, China, from January 2008 to April 2018.

Participants were included if they met the diagnostic criteria for TBM established by the expert consensus definition of 2010 [10]. According to the consensus definition, TBM patients were classified into three categories based on clinical information, CSF analysis, cerebral imaging, and evidence of tuberculosis elsewhere: definite TBM (microbiological identification or evidence from commercial nucleic acid amplification tests of CSF, or positive histological findings); probable TBM (diagnostic score of 10 or more points when cerebral imaging is not available, or 12 or above when cerebral imaging is available); and possible TBM (diagnostic score of 6–9 points when cerebral imaging is not available or 6–11 points when imaging is available). Patients were excluded if an alternative diagnosis was established, or if there were convincing signs of dual disease, or if a favorable outcome was observed in the absence of anti-tuberculosis therapy [10].

### Data collection

Data were collected following the standardized methods for enhanced quality and comparability of TBM study guidelines [11]. Mental status at admission was staged based on the modified MRC criteria as: stage I: the Glasgow Coma Scale (GCS) score of 15 and the absence of neurological deficit; stage II: GCS of 11–14, or GCS of 15 associated with focal neurological sign; and stage III: GCS ≤ 10 [12]. The health status at admission was assessed by the acute physiology and chronic health evaluation (APACHE) II and sequential organ failure assessment (SOFA) scores [13, 14]. Immune deficiency was considered in the case of human immunodeficiency virus (HIV) infection, solid cancer, hematological malignancy, diabetes mellitus, steroid therapy, and/or chemotherapy. Initial anti-tuberculosis therapy consisted of a standard regimen with four drugs: isoniazid, rifampicin, ethambutol, and pyrazinamide. Data on adjunctive steroids and the use of invasive mechanical ventilation and neurosurgical interventions (external ventricular drainage) during the ICU stay were collected.

### Outcomes

The primary endpoint was graded with the Glasgow Outcome Scale (GOS) 28 days after ICU admission as: 1: death; 2: vegetative state; 3: severe disability; 4: moderate disability; and 5: good recovery [15]. A favorable outcome was defined as GOS of 4–5, and an unfavorable outcome as GOS of 1–3 [16]. The second endpoint was overall mortality during a 1-year follow-up period.

### Statistical analysis

Data were presented as median (range) or number (%). Patients’ characteristics were compared according to primary outcomes, using Mann-Whitney tests for quantitative variables and Fisher’s exact tests for categorical variables. Durations were calculated from the time of ICU admission. Univariate logistic regression analysis was performed to evaluate the relationships between variables and primary outcome. Variables associated with unfavorable outcome in univariate analysis (*P* < 0.10) were included in the multivariate model. Discriminations among the different scoring systems were tested using the area under the receiver operating characteristic (ROC) curves. The best cut-off points were that which maximized the sum of sensitivity and specificity. Survival outcomes were measured by the Kaplan-Meier survival curve, and the log-rank test was used to determine statistical difference. Univariate Cox proportional hazard regression model was performed to evaluate associations between variables and 1-year mortality. Variables associated with mortality in univariate analysis (*p* < 0.10) were entered into the multivariate model. *P* < 0.05 was considered statistically significant. All analyses were conducted using SPSS 20.0 (IBM Inc., Armonk, NY, USA) software.

### Ethical approval

This study was approved by the ethics committee of Shandong Provincial Chest Hospital and informed consent was waived.

## Results

### Patients’ characteristics

Among the 151 patients with suspected TBM admitted to the ICU, 80 were included (Additional file 1). The baseline characteristics of the patients are summarized in Table 1. The median age was 38.5 (18–79) years and 45 (56 %) were males. All patients had MRC stage III illness on admission. HIV status was known in 71 and none were HIV-infected. Based on the consensus definition [10], 31 (39 %) patients were diagnosed with definite and probable TBM respectively, and 18 (22 %) patients with possible TBM. The median duration before ICU admission was 20 (3-365) days. Altered consciousness (87 %), lethargy (85 %), neck stiffness (83 %), fever (77 %) and headache (73 %) were the most common symptoms and signs.

**Table 1:** Baseline characteristics of the patients

Variables	All patients (*n* = 80)	Unfavorable outcome (*n* = 39)	Favorable outcome (*n* = 41)	*P* value
Demographic features				
Male sex	45 (56.3)	18 (46.2)	27 (65.9)	0.31
Age,years	38.5 (18–79)	44 (20–79)	36 (18–76)	0.31
Clinical features				
History of tuberculosis infection	7 (8.8)	5 (12.8)	2 (4.9)	0.26
Active extra-neural tuberculosis	41 (51.3)	20 (51.3)	21 (51.2)	> 0.99
Immune deficiency	15 (18.8)	10 (25.6)	5 (12.2)	0.16
Duration of symptoms, days	20 (3-365)	20 (6-365)	20 (3-365)	0.42
Length of ICU stay, days	12.5 (1-207)	9 (1-207)	15 (5-149)	0.03
Headache	59 (73.8)	31 (79.5)	28 (68.3)	0.31
Irritability	23 (28.8)	6 (15.4)	17 (41.5)	0.01
Nausea and vomiting	47 (58.8)	23 (59.0)	24 (58.5)	> 0.99
Fever (≥ 38.5℃)	60 (75.0)	28 (71.8)	32 (78.0)	0.61
Neck stiffness	67 (83.8)	32 (82.1)	35 (85.4)	0.77
Convulsions	15 (18.8)	9 (23.1)	6 (14.6)	0.40
Focal neurological deficits	43 (53.8)	20 (51.3)	23 (56.1)	0.82
Altered consciousness	70 (87.5)	33 (84.6)	37 (90.2)	0.51
Lethargy	68 (85.0)	32 (82.1)	36 (87.8)	0.54
GCS	3 (3–10)	3 (3–8)	3 (3–10)	0.06
APACHE II	23 (10–37)	26 (12–37)	20 (10–33)	0.003
SOFA	7 (3–13)	7 (4–13)	6 (3–11)	0.03
Laboratory results				
Positive culture in CSF	22 (27.8)	13 (34.2)^a^	9 (22.0)	0.32
Positive AFB in CSF	1 (1.3)	1 (2.8)^b^	0 (0.0)	0.47
Positive PCR in CSF	21 (26.9)	11 (29.7)^c^	10 (24.4)	0.62
CSF glucose, mmol/L	2.32 (0.33–12.60)	2.30 (0.33–12.60)	2.40 (0.40-5.00)	0.79
CSF/blood glucose ratio	0.32 (0.03–0.70)	0.33 (0.03–0.70)	0.30 (0.05–0.68)	0.82
CSF protein level, mg/L	1462 (299–3816)	1471 (299–3188)	1453 (464–3816)	0.31
CSF leukocyte, /µl	104 (2-1268)	108 (4-768)	102 (2-1268)	0.88
Peripheral blood leukocyte, /µl	10,045 (3420–40,400)	10,070 (3420–40,400)	9780 (3470–26,020)	0.36
Serum sodium, mmol/L	137 (107–158)	137 (117–158)	137 (107–154)	0.67
Cranial CT				
Hydrocephalus	45 (61.6)	25 (71.4) ^d^	20 (52.6) ^e^	0.15
Basal meningeal enhancement	10 (13.7)	5 (14.3) ^d^	5 (13.2) ^e^	> 0.99
Infarct	36 (49.3)	20 (57.1) ^d^	16 (42.1) ^e^	0.25
Tuberculoma	4 (5.5)	3 (8.6) ^d^	1 (2.6) ^e^	0.34
Pre-contrast basal hyperdensity	6 (8.2)	4 (11.4) ^d^	2 (5.3) ^e^	0.42
Mechanical ventilation	58 (72.5)	37 (94.9)	21 (51.2)	< 0.001
Surgical intervention	11 (13.8)	7 (17.9)	4 (9.8)	0.34

Data are presented as median (range) or numbers (percentages)

Abbreviations: *AFB* acid-fast bacilli, *APACHE* acute physiology and chronic health evaluation, *CSF* cerebrospinal fluid, *CT* computed tomography, *GCS* Glasgow coma scale, *ICU* intensive care unit, *PCR* polymerase chain reaction, *SOFA* sequential organ failure assessment

^a^Data missed in 1 case

^b^Data missed in 3 cases

^c^Data missed in 2 cases

^d^Data missed in 4 cases

^e^Data missed in 3 cases

CSF analysis revealed a typical pleocytosis of 104 (2-1268) cells/µl, elevated protein levels of 1462 (299–3816) mg/L, and low glucose levels of 2.32 (0.33–12.60) mmol/L. CSF cultures for *Mycobacterium tuberculosis* were positive in 22 (28 %) of the 79 patients’ CSF specimens on which the test were performed, including one multidrug-resistant and three rifampicin-resistant strains (Table 2). CSF polymerase chain reactions (PCR) were positive in 21 (27 %) of 78 patients. Among cranial images, hydrocephalus was found in 45 (62 %) of 73 patients while infarcts were found in 36 (49 %). The median scores of GCS, APACHE II and SOFA were 3 (3–10), 23 (10–37), and 7 (3–13) respectively on admission.

**Table 2:** CSF cultures for *Mycobacterium tuberculosis*

	Favorable	Unfavorable
Positive cultures, n/N	9/41	13/38^a^
Drug-susceptibility test results available, n	7^b^	10^c^
No resistance	6	4
Rifampicin resistance	0	3
Multidrug resistance	0	1
Resistant to others	1	2

Abbreviations: *CSF* cerebrospinal fluid

^a^Data missed in 1 case

^b^Data missed in 2 cases

^c^Data missed in 3 cases

First-line anti-tuberculosis therapy consisted of isoniazid (5–10 mg/kg, maximum 600 mg), rifampicin (10 mg/kg, maximum 600 mg), ethambutol (15 mg/kg, maximum 750 mg), and pyrazinamide (25 mg/kg, maximum 1500 mg) was initiated in all cases on admission. Four patients were secondarily detected with resistance to first-line drugs and switched to other drugs during their ICU stay. Adjunctive steroids were given to reduce inflammation in all patients on admission. Overall, 58 (73 %) patients received invasive mechanical ventilation and 11 (14 %) received lateral ventricular drainage during their ICU stay.

### Outcomes

**Fig. 1:**
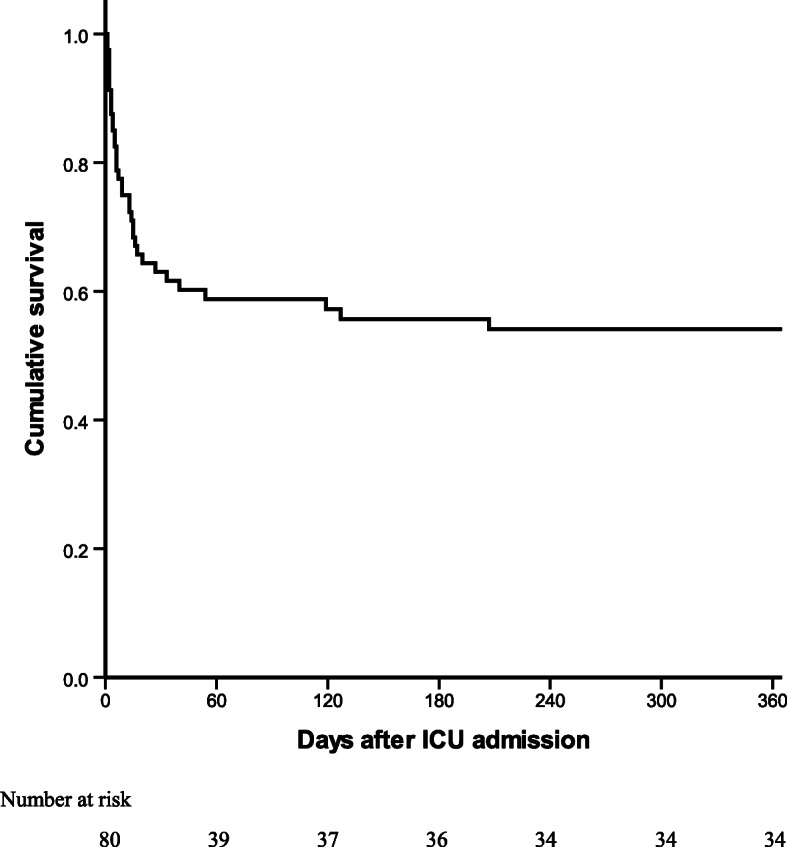
Kaplan-Meier estimates of overall survival at 1 year. ICU, intensive care unit

At 28 days, 39 (49 %) patients had unfavorable outcomes, including 29 (36 %) deaths. Variables including clinical and laboratory characteristics, and the three evaluating scores were independently analyzed in univariate logistic regression analysis. Only irritability, mechanical ventilation, APACHE II and SOFA scores showed statistical significance (Table 3). Multivariate logistic regression analysis identified three independent factors of unfavorable outcome (Table 4): APACHE II > 23 (adjusted odds ratio (aOR) 5.57, 95 % confidence interval (CI) 1.55–19.97), SOFA > 8 (aOR 9.74, 95 % CI 1.46–64.88), and the requirement of invasive mechanical ventilation (aOR 18.33, 95 % CI 3.15–106.80). One-year outcomes were available for 69 patients. Six patients died during the follow-up period, all of whom were from the unfavorable-outcome group. The 1-year overall mortality estimated by Kaplan-Meier analysis was 46 % (Fig. 1). The univariate Cox regression analysis is presented in Table 5. Multivariate Cox regression analysis identified two factors positively associated with 1-year mortality (Table 6): APACHE II > 23 (adjusted hazard ratio (aHR) 4.83; 95 % CI 2.21–10.55), and the requirement of mechanical ventilation (aHR 9.71; 95 % CI 2.31–40.87). Among 1-year survivors, functional independence (GOS of 5) was observed in 28/34 (82 %) cases. Of the 6 patients with functional dependence (GOS of 4), visual impairment was found in one case and reduced muscle strength was found in five cases.

**Table 3:** Factors associated with outcome by univariate logistic regression analysis

Variable	Unfav	Fav	OR (95 % CI)	*P* Value	Variable	Unfav	Fav	OR (95 % CI)	*P* Value
Sex					Headache				
Male	18	27	0.44 (0.18–1.10)	0.08	Yes	31	28	1.80 (0.65–4.98)	0.26
Female	21	14			No	8	13		
Age, years					Irritability				
> 60	10	6	2.01 (0.65–6.20)	0.22	Yes	6	17	0.26 (0.09–0.75)	0.01
≤ 60	29	35			No	33	24		
Duration of Symptoms, days					Nausea and vomiting				
> 10	32	30	1.68 (0.58–4.89)	0.34	Yes	23	24	1.02 (0.42–2.48)	0.97
≤ 10	7	11			No	16	17		
ICU stay, days					Fever(≥ 38.5℃)				
> 15	12	18	0.57 (0.23–1.42)	0.23	Yes	28	32	0.72 (0.26–1.98)	0.52
≤ 15	27	23			No	11	9		
Immune deficiency					Neck stiffness				
Yes	10	5	2.48 (0.76–8.08)	0.13	Yes	32	35	0.78 (0.24–2.58)	0.69
No	29	36			No	7	6		
Old tuberculosis					Convulsions				
Yes	5	2	2.87 (0.52–15.75)	0.23	Yes	9	6	1.75 (0.56–5.49)	0.34
No	34	39			No	30	35		
Active extra-neuraltuberculosis					Focal neurologicaldeficits				
Yes	20	21	0.95 (0.39–2.30)	0.91	Yes	20	23	1.21 (0.50–2.93)	0.67
No	19	20			No	19	18		
Alteredconsciousness					Basal meningealenhancement				
Yes	33	37	0.60 (0.15–2.29)	0.45	Yes	5	5	1.10 (0.29–4.18)	0.89
No	6	4			No	30	33		
Lethargy					Hydrocephalus				
Yes	32	36	0.64 (0.18–2.20)	0.47	Yes	25	20	2.25 (0.85–5.94)	0.10
No	7	5			No	10	18		
GCS					Infarct				
≤ 4	32	29	1.89 (0.66–5.45)	0.24	Yes	20	16	1.83 (0.72–4.64)	0.20
> 4	7	12			No	15	22		
APACHE II					Tuberculoma				
≤ 23	13	29	0.21 (0.08–0.53)	0.001	Yes	3	1	3.47 (0.34–35.02)	0.29
> 23	26	12			No	32	37		
SOFA					MV				
≤ 8	23	39	0.07 (0.02–0.35)	0.001	Yes	37	21	17.62(3.74–82.93)	< 0.001
> 8	16	2			No	2	20		
Positive culture inCSF					Pre-contrast basalhyperdensity				
Yes	13	9	1.85 (0.68–5.02)	0.23	Yes	4	2	2.32 (0.40-13.55)	0.35
No	25	32			No	31	36		
Positive PCR in CSF					Surgical intervention				
Yes	11	10	1.31 (0.48–3.57)	0.60	Yes	7	4	2.02 (0.54–7.55)	0.29
No	26	31			No	32	37		

Abbreviations: *APACHE* acute physiology and chronic health evaluation, *CI* confidence interval, *CSF* cerebrospinal fluid, *Fav* favorable, *GCS* Glasgow coma scale, *ICU* intensive care unit, *MV* mechanical ventilation, *OR* odds ratio, *PCR* polymerase chain reaction, *SOFA *sequential organ failure assessment, *Unfav* unfavorable

**Table 4:** Prognostic factors of unfavorable outcome by multivariate logistic regression

Variable	OR	95 % CI	*P* Value
APACHE II > 23	5.57	1.55–19.97	0.008
SOFA > 8	9.74	1.46–64.88	0.019
Mechanical ventilation	18.33	3.15–106.80	0.001

Abbreviations: *APACHE* acute physiology and chronic health evaluation, *CI* confidence interval, *OR* odds ratio, *SOFA* sequential organ failure assessment

**Table 5:** Univariate Cox regression analysis of factors associated with 1-year mortality

Variable	HR	95 %CI	*P* value
Age	1.019	1.00-1.04	0.055
Male sex	0.470	0.24–0.92	0.027
Irritability	0.934	0.48–1.82	0.841
GCS	1.430	0.62–3.28	0.398
APACHEII	4.632	2.15–9.99	< 0.001
SOFA	3.646	1.85–7.19	< 0.001
MV	9.331	2.23–39.04	0.002

Abbreviations: *HR* hazard ratio, *CI* confidence interval, *GCS* Glasgow coma scale, *APACHE* acute physiology and chronic health evaluation, *SOFA* sequential organ failure assessment, *MV* mechanical ventilation

**Table 6:** Multivariate Cox analysis of factors associated with 1-year mortality

Variable	HR	95 % CI	*P* value
APACHE II > 23	4.83	2.21–10.55	< 0.001
Mechanical ventilation	9.71	2.31–40.87	0.002

Abbreviations: *HR* hazard ratio, *CI* confidence interval, *APACHE* acute physiology and chronic health evaluation

**Fig. 2:**
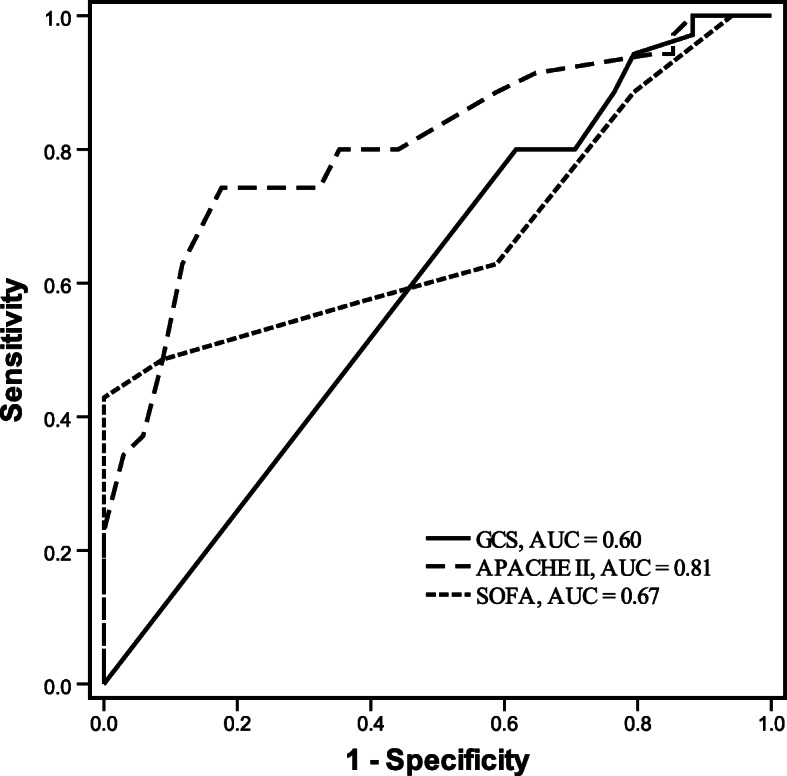
ROC curves for GCS, APACHE II and SOFA. ROC, receiver operating characteristic; GCS, Glasgow Coma Scale; APACHE, Acute Physiology and Chronic Health Evaluation; SOFA, Sequential Organ Failure Assessment; AUC, area under the curve

**Fig. 3:**
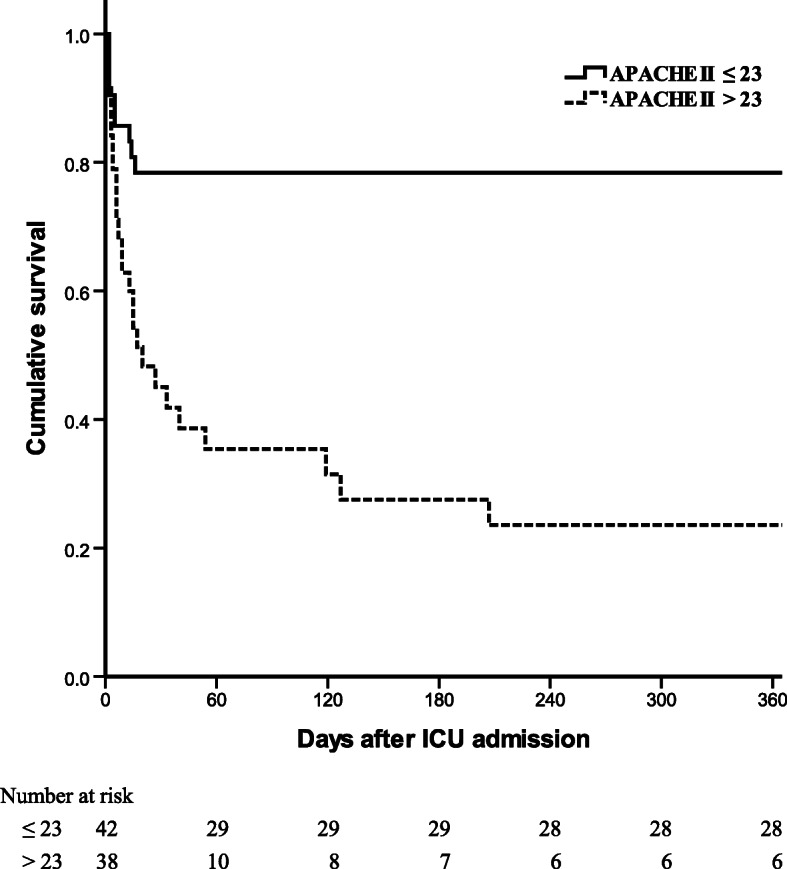
Kaplan-Meier survival curves of patients with APACHE II ≤ 23 and > 23. *P *(log rank test) < 0.001. APACHE, Acute Physiology and Chronic Health Evaluation; ICU, intensive care unit

In the ROC analysis (Fig. 2), the areas under the curve were: GCS 0.60 (95 % CI 0.46–0.73; *P* = 0.17), APACHE II 0.81 (95 % CI 0.70–0.91; *P* < 0.001), and SOFA 0.67 (95 % CI 0.54–0.80; *P* = 0.01). To obtain the strongest power of prediction, the cut-off points were 4 for GCS (sensitivity 0.80 and specificity 0.38), 23 for APACHE II (sensitivity 0.74 and specificity 0.82), and 8 for SOFA (sensitivity 0.43 and specificity 1.00) respectively. Kaplan-Meier survival curves for patients with APACHE II score ≤ 23 and > 23 are shown in Fig. 3, with *P* < 0.001. Patients stratified by SOFA (≤ 8 and > 8) yielded the similar result (*P* < 0.001; Additional file 2). There is no statistically significant difference between survival curves for patients stratified by GCS ≤ 4 and > 4 (*P* = 0.38; Additional file 3).

When APACHE II,SOFA and mechanical ventilation were included in a model, the model showed good discrimination as evident by an AUC = 0.878 (95 % CI 0.805–0.950, *P* < 0.001; Additional file 4) and good calibration (Hosmer and Lemeshow test *P* = 0.904; Additional file 5).

## Discussion

Our study found that both the APACHE II and SOFA scores on admission predicted short-term outcomes of adult patients with TBM in ICU. And the APACHE II scoring system was superior to SOFA in predicting 1-year outcomes. In addition, the requirement of invasive mechanical ventilation was found to be independently associated with an unfavorable outcome.

In our study, the participants’ characteristics differed significantly from those reported in previous studies conducted in other endemic areas [17, 18], but were similar to the one conducted in non-endemic area [4]. Most obviously, all the 80 patients presented with MRC stage III illness, and 58 (73 %) required invasive mechanical ventilation. These differences might be explained by selection bias of the most severe cases requiring ICU admission in this study, since the access to intensive care was low in China. Different factors such as older age, hydrocephalus, change in consciousness and higher MRC stage were reported to be associated with poor prognosis in patients with TBM [4, 19–21]. However, no clinical, laboratory or imaging factors were found to be associated with poor outcomes in our study cohort. This could be explained by the participants themselves, since they were the most severe cases who were more likely to develop hydrocephalus and comatose than those with MRC stage I or II illness.

Thirty-five patients in our cohort died, among whom 29 deaths were within 28 days. The 1-year overall mortality was 46 %, which was extremely high, but comparable to a previous study [5]. In that systematic review and meta-analysis, the mortality rate was 64.8 % for patients with stage III TBM [5]. The phenomenon that most deaths (29/35) occurred early was consistent with a Madagascar cohort and a meta-analysis which consisted of 5752 adult TBM patients [22, 23].

Mechanical ventilation was reported to be required by 10-20 % of adult patients with TBM in all stages [24, 25]. For those admitted to ICU, this number increased to 70 % [4, 26]. In our study, 58 (73 %) patients received invasive mechanical ventilation during their ICU stay, of whom 37 (64 %) had an unfavorable outcome. Consistent with previous studies, the requirement of mechanical ventilation was associated with an unfavorable outcome [24, 25]. Those who needed mechanical ventilation were more critically ill because of associated sepsis and TBM-related or systemic complications, and had a higher mortality rate.

Hyponatremia is the most common electrolyte abnormality observed in hospitalized individuals and is associated with increased mortality [27]. For patients with TBM, the frequency of hyponatremia was reported to be about 40–50 % in different studies [28–30]. The relationship between hyponatremia and death was uncertain. It was reported to be certain in a tertiary care cohort [30]. However, hyponatremia didn’t have a predictive value on the outcome of TBM in another prospective study [29]. In a study consisted of 1048 adult TBM patients, the authors used time-updated Glasgow coma score and plasma sodium measurements to dynamically predict the death, they found that plasma sodium values were higher in HIV-infected survivors, with a less clear relationship between sodium and survival in HIV-uninfected patients [31]. We didn’t observe an association of plasma sodium levels with death in our study cohort, too. So, more prospective studies need to be carried out in different patient populations to confirm the role of hyponatremia in TBM.

GCS was used to assess the mental status of patients with TBM and low GCS scores were reported to be associated with an unfavorable outcome in numerous studies [32–35]. In our study, GCS had no association with unfavorable outcomes, which might be explained by the relatively lower GCS scores of the patients with MRC stage III illness on admission. Irritability displayed a better association with favorable outcomes in univariate analysis, partially due to its relationship with a relatively higher GCS score.

APACHE II and SOFA were the most common used scoring systems to evaluate the disease severity of patients in ICU [13, 14]. To our knowledge, few studies have used APACHE II in patients with TBM [16], and none has used SOFA. In the previous study, APACHE II showed a good predictive value as GCS and superior to MRC for discharge outcomes of adult patients with TBM [16]. To obtain the greatest power in prediction based on ROC curve analysis, the cut-off points chosen for GCS, APACHE II and SOFA were 4, 23 and 8 respectively in our study. The results showed that only APACHE II had both acceptable sensitivity and specificity. In univariate and multivariate logistic regression analyses, APACHE II and SOFA were independently associated with an unfavorable outcome. APACHE II > 23 was identified as predictor of 1-year mortality by multivariate Cox regression analysis. These two scoring systems were based on physiological variables other than levels of consciousness or neurological deficits, on which the GCS was based. Therefore the use of APACHE II and SOFA would be more suitable for assessing the prognosis of patients with TBM, especially for those admitted to ICU.

Imran and collegues have derived a simple bedside score (MASH-P) including variables baseline modified Barthel index (M), age (A), stage (S), hydrocephalus (H) and papilledema (P), which can be used easily at bedside to predict 6-month mortality in tuberculous meningitis [36]. However, the model needs external validation to assess its performance in different settings. In our study cohort, a model including APACHE II, SOFA and mechanical ventilation also showed good discrimination and good calibration. However, it also needs external validation and further assessment since there were duplicate indices between the two scoring systems such as GCS scores, mean arterial pressure and creatinine. APACHE II itself had a good predictive value on the outcome of TBM (area under the ROC curve = 0.81), so we would suggest using APACHE II alone instead of the three-factor model to reduce the workload of clinicians.

Our study has some strengths. First, no study was conducted on adult patients with TBM admitted to ICU in China. Second, participants included in our study were the most severe TBM cases of MRC stage III - a group which was not previously reported separately. Moreover, we used validated guidelines and consensus definitions to include participants and report data.

Our study was limited by its retrospective design. All data were collected from a single medical center and the sample size was not large enough. Most patients only took cranial CT scans, which lack sensitivity for TBM-associated cerebrovascular or inflammatory complications. The present results may not be applied to a less severe population since we focused on the most severe TBM cases of stage III admitted to the ICU.

## Conclusions

For the most severe adult TBM patients of MRC stage III, common clinical factors aren’t effective enough to predict outcomes. Our study demonstrates that the widely used APACHE II and SOFA scores on admission can be used to predict short-term outcomes, while APACHE II could also be used to predict long-term outcomes of adult patients with TBM in ICU.

## Supplementary Information

**Additional file 1: Figure S1.** The flow diagram of patient enrollment.

**Additional file 2: Figure S2.** Kaplan-Meier survival curves of patients with SOFA ≤ 8 and > 8.

**Additional file 3: Figure S3.** Kaplan-Meier survival curves of patients with GCS ≤ 4 and > 4.

**Additional file 4: Figure S4.** ROC curves for the final model including APACHE II, SOFA and mechanical ventilation.

**Additional file 5: Figure S5.** The calibration plot of the final model.

**Additional file 6.** Data of the research.

## Data Availability

The datasets used and/or analysed during the current study are available from the corresponding author on reasonable request.

## Abbreviations

TBM: Tuberculous meningitis
MRC: Medical Research Council
CSF: Cerebrospinal fluid
ICU: Intensive care unit
GCS: Glasgow Coma Scale.
APACHE: Acute Physiology and Chronic Health Evaluation
SOFA: Sequential Organ Failure Assessment
HIV: Human immunodeficiency virus
GOS: Glasgow Outcome Scale
ROC: Receiver operating characteristic
OR: Odds ratio
CI: Confidence interval
HR: Hazard ratio
