# Correction to: Prognostic factors of adult tuberculous meningitis in intensive care unit: a single-center retrospective study in East China

**DOI:** 10.1186/s12883-021-02433-z

**Published:** 2021-10-25

**Authors:** Baobao Feng, Xiao Fei, Ying Sun, Xingguo Zhang, Deya Shang, Yi Zhou, Meiyan Sheng, Jiarui Xu, Wei Zhang, Wanhua Ren

**Affiliations:** 1 Department of Emergency, Shandong Provincial Hospital, Cheeloo College of Medicine, Shandong University, Jinan, 250021 Shandong China; 2 Department of Emergency, Shandong Provincial Hospital Affiliated to Shandong First Medical University, Jinan, 250021 Shandong China; 3 Department of Infectious Diseases, Weifang Yidu Central Hospital, Weifang, 262500 Shandong China; 4 Department of Critical Care Medicine, Shandong Provincial Chest Hospital, Cheeloo College of Medicine, Shandong University, Jinan, 250013 Shandong China; 5 Department of Infectious Diseases, Shandong Provincial Hospital, Cheeloo College of Medicine, Shandong University, 324 Jingwu Weiqi Road, Jinan, 250021 Shandong China; 6 Department of Infectious Diseases, Shandong Provincial Hospital Affiliated to Shandong First Medical University, Jinan, 250021 Shandong China

**Correction to: BMC Neurol 21, 308 (2021)**

**https://doi.org/10.1186/s12883-021-02340-3**

Following publication of the original article [1], the authors reported errors in affiliations 1, 4 and 5.

Affiliation 1: Instead of “Department of Emergency, **Cheeloo College of Medicine, Shandong Provincial Hospital**, Shandong University, Jinan, Shandong, 250021, China”, it should be “Department of Emergency, **Shandong Provincial Hospital, Cheeloo College of Medicine**, Shandong University, Jinan, Shandong, 250021, China”.

Affiliation 4: Instead of “Department of Critical Care Medicine, **Cheeloo College of Medicine, Shandong Provincial Chest Hospital**, Shandong University, Jinan, Shandong, 250013, China”, it should be “Department of Critical Care Medicine, **Shandong Provincial Chest Hospital, Cheeloo College of Medicine**, Shandong University, Jinan, Shandong, 250013, China”.

Affiliation 5: Instead of “Department of Infectious Diseases, **Cheeloo College of Medicine, Shandong Provincial Hospital**, Shandong University, Jinan, Shandong, 250021, China”, it should be “Department of Infectious Diseases, **Shandong Provincial Hospital, Cheeloo College of Medicine**, Shandong University, Jinan, Shandong, 250021, China”.

The authors apologize for these errors and state that this does not change the scientific conclusions of the article in any way.

The original article [1] has been updated.
