# Supplementary material for: Prognostic factors of adult tuberculous meningitis in intensive care unit: a single-center retrospective study in East China
Source: BMC Neurol. 2021 Aug 10;21:308. doi: 10.1186/s12883-021-02340-3 (PMC8353730; doi:10.1186/s12883-021-02340-3)
Supplement: Supplementary file 1 — Additional file 1: Figure S1. The flow diagram of patient enrollment. [file 12883_2021_2340_MOESM1_ESM.doc]

**39** had unfavorable outcome

**151** patients with suspected TBM assessed for eligibility

**100** patients diagnosed with TBM

**80** patients fulfilled inclusion criteria

**41** had favorable outcome

**51** Excluded

**35** Because of alternative diagnosis

**16** Because of lack of CSF analysis

**20** Excluded because they were

younger than 18 years

**Figure S1. The flow diagram of patient enrollment.** TBM, tuberculous meningitis;

CSF, cerebrospinal fluid.
