# Supplementary material for: Prognostic factors of adult tuberculous meningitis in intensive care unit: a single-center retrospective study in East China
Source: BMC Neurol. 2021 Aug 10;21:308. doi: 10.1186/s12883-021-02340-3 (PMC8353730; doi:10.1186/s12883-021-02340-3)
Supplement: Supplementary file 2 — Additional file 2: Figure S2. Kaplan-Meier survival curves of patients with SOFA ≤ 8 and > 8. [file 12883_2021_2340_MOESM2_ESM.doc]

Number at risk

≤ 8 62 36 35 35 34 34 34

> 8 18 3 2 1 0 0 0

**Figure S2. Kaplan-Meier survival curves of patients with SOFA ≤ 8 and > 8.** *P* (log rank test) < 0.001. SOFA, Sequential Organ Failure Assessment; ICU, intensive care unit.
