# Supplementary material for: Prognostic factors of adult tuberculous meningitis in intensive care unit: a single-center retrospective study in East China
Source: BMC Neurol. 2021 Aug 10;21:308. doi: 10.1186/s12883-021-02340-3 (PMC8353730; doi:10.1186/s12883-021-02340-3)
Supplement: Supplementary file 3 — Additional file 3: Figure S3. Kaplan-Meier survival curves of patients with GCS ≤ 4 and > 4. [file 12883_2021_2340_MOESM3_ESM.doc]

Number at risk

≤ 4 61 28 26 25 24 24 24

> 4 19 11 11 11 10 10 10

**Figure S3. Kaplan-Meier survival curves of patients with GCS ≤ 4 and > 4.** *P* (log rank test) = 0.38. GCS, Glasgow Coma Scale; ICU, intensive care unit.
