# Supplementary material for: Prognostic factors of adult tuberculous meningitis in intensive care unit: a single-center retrospective study in East China
Source: BMC Neurol. 2021 Aug 10;21:308. doi: 10.1186/s12883-021-02340-3 (PMC8353730; doi:10.1186/s12883-021-02340-3)
Supplement: Supplementary file 4 — Additional file 4: Figure S4. ROC curves for the final model including APACHE II, SOFA and mechanical ventilation. [file 12883_2021_2340_MOESM4_ESM.doc]

**Figure S4 ROC curves for the final model including APACHE II, SOFA and mechanical ventilation.** Area under the curve = 0.878 (95% CI 0.805-0.950, *P* < 0.001). APACHE, Acute Physiology and Chronic Health Evaluation; ROC, receiver operating characteristic; SOFA, Sequential Organ Failure Assessment.
