# Supplementary figures and images for: Prognostic factors of adult tuberculous meningitis in intensive care unit: a single-center retrospective study in East China
Source: BMC Neurol. 2021 Aug 10;21:308. doi: 10.1186/s12883-021-02340-3 (PMC8353730; doi:10.1186/s12883-021-02340-3)

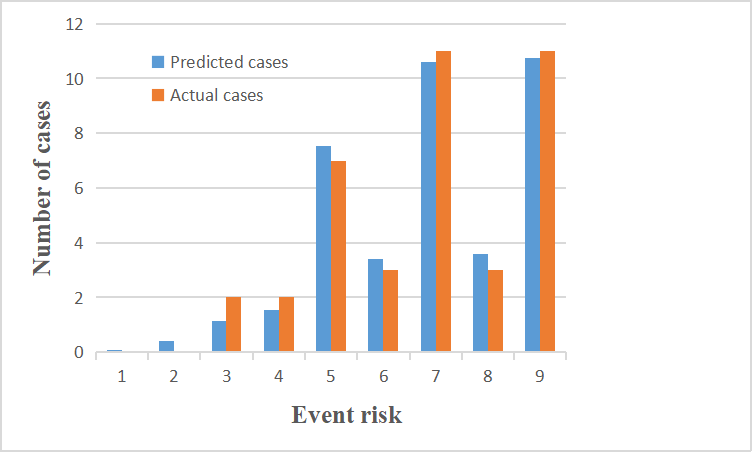


**Figure S5 The calibration plot of the final model.**

Supplement: Supplementary file 5 — Additional file 5: Figure S5. The calibration plot of the final model. [file 12883_2021_2340_MOESM5_ESM.docx]
